# Comorbidities in Patients with Psoriatic Arthritis

**DOI:** 10.5041/RMMJ.10279

**Published:** 2017-01-30

**Authors:** Amir Haddad, Devy Zisman

**Affiliations:** 1 Rheumatology Unit, Carmel Medical Centre, Haifa, Israel; 2 The Ruth and Bruce Rappaport Faculty of Medicine, Technion–Israel Institute of Technology, Haifa, Israel

**Keywords:** Comorbidity, management, psoriatic arthritis

## Abstract

Epidemiological studies have shown that patients with psoriatic arthritis (PsA) are often affected by numerous comorbidities that carry significant morbidity and mortality. Reported comorbidities include diabetes mellitus, obesity, metabolic syndrome, cardiovascular diseases, osteoporosis, inflammatory bowel disease, autoimmune eye disease, non-alcoholic fatty liver disease, depression, and fibromyalgia. All health care providers for patients with PsA should recognize and monitor those comorbidities, as well as understand their effect on patient management to ensure an optimal clinical outcome.

## INTRODUCTION

Psoriatic arthritis (PsA) is a heterogeneous multifaceted inflammatory arthritis associated with psoriasis; it is considered to be one of the spondyloarthritides and as such has both spinal and peripheral joint involvement as well as enthesitis and dactylitis. In addition to the joint and skin manifestations, PsA is associated with numerous extra-articular immune-mediated manifestations such as inflammatory bowel disease and autoimmune ophthalmic disease. Studies have shown that patients with psoriatic disease suffer also from associated comorbidities, including cardiovascular disease, obesity and metabolic syndrome, diabetes, osteoporosis, malignancy, fatty liver disease, depression, and anxiety.^1^ To ensure an optimal outcome, identifying these comorbidities is of utmost importance. The objectives of this review are to present and discuss the available evidence on comorbidities in PsA patients.

## CARDIOVASCULAR DISEASE

Cardiovascular disease (CVD) is one of the most significant comorbidities in rheumatic diseases in general, and in psoriatic disease in particular, where the systemic inflammation leads to increased insulin resistance, endothelial cell dysfunction, and the development of atherosclerosis.^2^ A meta-analysis of 75 observational studies found that psoriasis is associated with a relative risk of 1.4 (95% CI 1.2–1.7) for CVD.^3^ Although there are fewer studies on cardiovascular risk in PsA compared with that in psoriasis, several studies have shown a similar trend.^4^,^5^ A recent population-based cohort study has shown that the risk of major adverse cardiovascular events was higher in PsA patients not prescribed a disease-modifying antirheumatic drug (DMARD) (HR 1.24, 95% CI 1.03–1.49) compared to the general population after adjusting for traditional cardiovascular risk factors but without increase in mortality.^6^ The association was found to be independent of traditional CVD risk factors such as hypertension, dyslipidemia, and smoking and correlated with markers of disease severity and activity,^7^ suggesting that optimal treatment of the disease would improve CVD outcomes. To date, however, as far as we could find in a far-ranging review of the literature, no study has specifically examined the effect of aggressive PsA treatment regimens on the risk of cardiovascular events. On the other hand, studies on patients with rheumatoid arthritis and psoriasis have shown a reduced rate of cardiovascular events among patients treated with anti-tumor necrosis factor alpha (TNFα) medications.^8^,^9^ In patients with comorbid CVD, the use of non-steroidal anti-inflammatory drugs should be at the lowest effective dose for the shortest period of time possible.^10^

## DIABETES MELLITUS, METABOLIC SYNDROME, AND OBESITY

Diabetes mellitus, metabolic syndrome, and obesity were reported to be at increased prevalence in many studies on patients with psoriatic arthritis, with a crude OR of 2.18 (95% CI 1.36–3.50) of type 2 diabetes mellitus in PsA, and patients with severe psoriasis having a higher risk.^11^–^13^ Among diabetic patients, psoriasis is generally associated with higher rates of microvascular and macrovascular complications.^14^ Patients with PsA have a higher BMI compared to rheumatoid arthritis patients and the general population.^15^ In patients with PsA, metabolic syndrome and insulin resistance are highly prevalent and were found to be independently associated with the severity of underlying PsA.^16^ Several mechanisms could explain the association between PsA and diabetes, such as patients’ unhealthy lifestyle,^17^ the inflammatory cytokine milieu that drives insulin resistance,^18^–^20^ as well as shared genetic loci for susceptibility to psoriasis and diabetes.^21^–^23^ A large study on patients with PsA conducted in Israel also found an association with diabetes even after controlling for potential confounders, including age, obesity, and steroid treatment.^24^ This finding might also have therapeutic implications, as ongoing studies are investigating the effect of antidiabetic drugs on psoriasis.^25^,^26^

## OSTEOPOROSIS

Osteoporosis was reported in studies on patients with various inflammatory rheumatic diseases,^27^–^30^ as well as increased risk for low bone density and fragility fractures.^31^ Skeletal manifestations of PsA are complex and comprise both new bone formation manifesting with bone ankylosis, periostitis, and syndesmophytes, and bone resorption in the form of erosions. The prevalence of osteoporosis in PsA has not been studied to the same degree. The literature review with regard to bone mineral density in PsA shows inconsistent and conflicting results.^32^–^34^ Patients with PsA in Israel, however, were also found to be at increased risk of osteoporosis.^24^

## INFLAMMATORY BOWEL DISEASE

Inflammatory bowel disease (IBD) as well as subclinical bowel inflammation have been observed with increased incidence in patients with psoriasis, and a pronounced risk was found in patients with concomitant PsA (RR 6.43, 95% CI 2.04–20.32) for Crohn’s disease but not for ulcerative colitis.^35^,^36^ Occasionally, patients may develop a paradoxical IBD when being treated with an anti-TNFα agent.^37^ One debatable issue is whether non-steroidal anti-inflammatory drugs (NSAIDs) may exacerbate IBD symptoms.^38^ No data have been published assessing the appropriate therapy for concomitant PsA and IBD, although common medications are being used to treat both conditions. Among the anti-TNFα medications, the monoclonal TNFα receptor etanercept was not shown to be effective in treatment of IBD.

## AUTOIMMUNE OPHTHALMIC DISEASE

Autoimmune ophthalmic diseases, including uveitis, keratitis, blepharitis, conjunctivitis, episcleritis, and scleritis, have been observed,^39^–^41^ with uveitis being the most common.^42^ Autoimmune ophthalmic disease often precedes PsA or progresses despite adequate therapy for PsA. As in the management of inflammatory bowel disease, etanercept may not adequately control the uveitis.^43^ Moreover, as the case numbers are low, a causative role cannot be discerned with the use of anti-TNFα agents in PsA patients who develop uveitis.^44^

## MALIGNANCY

Malignancies increased in patients with systemic autoimmune rheumatic diseases, particularly rheumatoid arthritis,^45^ systemic lupus erythematosus,^46^ systemic sclerosis,^47^ and idiopathic inflammatory myopathies.^48^ Studies on patients with PsA, however, did not show an increased risk of solid malignancies.^49^ In a meta-analysis of randomized controlled trials (RCTs) across all indications, short-term use of TNFα inhibitors was not associated with a significantly increased risk of cancer.^50^ No statistically significant increase in non-melanoma skin cancers was observed in patients using TNFα blockers.^50^

## LIVER DISEASE

Liver disease, particularly non-alcoholic fatty liver disease (NAFLD), has an increased prevalence in patients with psoriasis.^51^ Few studies have examined this relationship solely in PsA patients.^52^,^53^ Among patients with psoriasis, however, an increased prevalence of NAFLD is associated with metabolic syndrome, hypercholesterolemia, hypertriglyceridemia, obesity, psoriasis severity, and concomitant PsA.^54^ The use of medications such as NSAIDs, DMARDs, and TNFα blockers may be also associated with liver function test (LFT) abnormalities and hepatotoxicity. Higher rates of non-alcoholic steatohepatitis (NASH)/NAFLD may occur in PsA patients using methotrexate compared to rheumatoid arthritis (RA),^55^ and LFT abnormalities may be similar or slightly higher in PsA patients.^52^,^53^,^56^ Studies of psoriasis have shown that the development of NASH/NAFLD in long-term users of methotrexate was associated with cumulative doses of methotrexate as well as presence of obesity or diabetes.^57^ So patients should be monitored regularly for liver function tests abnormalities; a liver biopsy should be considered, based on the presence or absence of risk factors for hepatotoxicity and cumulative methotrexate dose.^58^

## DEPRESSION AND ANXIETY

Depression and anxiety could be related to the impact of psoriasis, arthritis, or inflammation.^59^ It is estimated to affect more than 30% of psoriasis patients.^60^ The rate of depression and anxiety was reported to be significantly higher in patients with PsA than in those with psoriasis and was associated with disease-related factors such as actively inflamed joint count as well as disability, pain, and fatigue.^61^ Treatment with TNFα blockers was associated with reduced rates of depression and insomnia, as well as a reduced rate of regular antidepressant use.^62^

## FIBROMYALGIA

Coexisting fibromyalgia should be identified and differentiated from enthesitis. Fibromyalgia was reported in 53% of patients in one study.^63^ It is related to worse patient-reported outcomes and disease activity measures. The influence of coexisting fibromyalgia should be taken into consideration in the treatment decision, in order to avoid unnecessary treatment escalation.^64^

## CONCLUSION

In this review we have highlighted some important comorbid diseases and extra-articular manifestations that can have an impact on patient care, management, treatment decisions, and quality of life as well as mortality. We recommend that, to provide comprehensive medical care to patients with PsA, physicians should be aware of these comorbid disease associations.

## Abbreviations

CVD: cardiovascular disease
DMARD: disease-modifying antirheumatic drug
IBD: inflammatory bowel disease
LFT: liver function test
NAFLD: non-alcoholic fatty liver disease
NASH: non-alcoholic steatohepatitis
NSAID: non-steroidal anti-inflammatory drug
PsA: psoriatic arthritis
TNFα: tumor necrosis factor alpha
